# Airway basal stem cells generate distinct subpopulations of PNECs

**DOI:** 10.1016/j.celrep.2021.109011

**Published:** 2021-04-20

**Authors:** Hongmei Mou, Ying Yang, Molly A. Riehs, Juliana Barrios, Manjunatha Shivaraju, Adam L. Haber, Daniel T. Montoro, Kimberly Gilmore, Elisabeth A. Haas, Brankica Paunovic, Jayaraj Rajagopal, Sara O. Vargas, Robin L. Haynes, Alan Fine, Wellington V. Cardoso, Xingbin Ai

**Affiliations:** 1 The Mucosal Immunology and Biology Research Center, Massachusetts General Hospital for Children, Boston, MA 02114, USA; 2 Columbia Center for Human Development and Pulmonary Allergy & Critical Care Medicine, Department of Medicine, Columbia University Medical Center, New York, NY 10032, USA; 3 Department of Pathology, Boston Children’s Hospital, MA 02115, USA; 4 Center for Regenerative Medicine, Massachusetts General Hospital, Boston, MA 02114, USA; 5 Computational Biology and Environmental Health, Harvard T.H. Chan School of Public Health, Boston, MA 02115, USA; 6 Klarman Cell Observatory, Broad Institute of MIT and Harvard, Cambridge, MA 02142, USA; 7 Division of Neonatology and Newborn Medicine, Department of Pediatrics, Massachusetts General Hospital, Boston, MA 02114, USA; 8 Department of Research, Rady Children’s Hospital, San Diego, CA 92123, USA; 9 San Diego County Office of the Medical Examiner, San Diego, CA 92123, USA; 10 Pulmonary Division, Boston University School of Medicine, Boston, MA 02118, USA; 11 Lead contact

## Abstract

Pulmonary neuroendocrine cells (PNECs) have crucial roles in airway physiology and immunity by producing bioactive amines and neuropeptides (NPs). A variety of human diseases exhibit PNEC hyperplasia. Given accumulated evidence that PNECs represent a heterogenous population of cells, we investigate how PNECs differ, whether the heterogeneity is similarly present in mouse and human cells, and whether specific disease involves discrete PNECs. Herein, we identify three distinct types of PNECs in human and mouse airways based on single and double positivity for TUBB3 and the established NP markers. We show that the three PNEC types exhibit significant differences in NP expression, homeostatic turnover, and response to injury and disease. We provide evidence that these differences parallel their distinct cell of origin from basal stem cells (BSCs) or other airway epithelial progenitors.

## In brief

Mou et al. find that the conducting airway harbors three distinct types of pulmonary neuroendocrine cells, distinguished by gene expression, cell of origin, and existence in solitary and clustered configurations. The expansion of PNEC types is selectively associated with NEHI and SIDS, suggesting their contribution to disease.

## Graphical Abstract

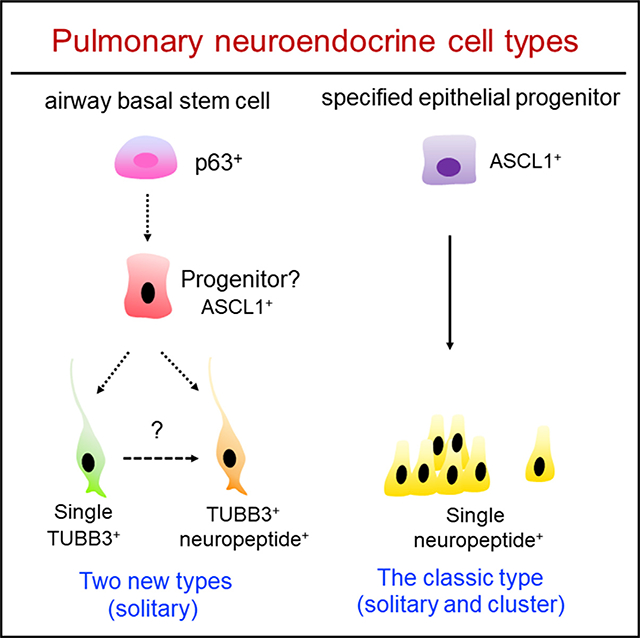

## INTRODUCTION

Pulmonary neuroendocrine cells (PNECs) comprise a rare population of epithelial cells in mammalian airways that uniquely express bioactive amines and neuropeptides (NPs), including calcitonin gene-related peptide (CGRP), gastrin-releasing peptide (GRP), and chromogranin A (CHGA) (Gosney et al., 1988; Weichselbaum et al., 2005). These cells are crucial components of the innate immunity in airways, responding to environmental stimuli by releasing NPs and neurotransmitters to mediate a variety of functions.

PNECs can be found as solitary cells and in clusters (neuroepithelial bodies [NEBs]). PNECs are mostly studied in the configuration of NEBs that are found preferentially at airway bifurcations and bronchioalveolar duct junctions in animal models. This differs from humans, in which PNECs are mostly solitary and randomly distributed along the airways (Gosney et al., 1988; Weichselbaum et al., 2005). Human and mouse PNECs also differ in the expression of amines and the NP marker. For example, CGRP and γ-aminobutyric acid (GABA) are uniformly expressed by solitary PNECs and NEBs in the mouse intrapulmonary airway (Schnorbusch et al., 2013; Barrios et al., 2017). However, these two markers are detected in only a fraction of PNECs in humans and nonhuman primates (Gosney et al., 1988; Weichselbaum et al., 2005; Barrios et al., 2019). NEBs and the total number of PNECs are markedly increased in a variety of human diseases, such as neuroendocrine hyperplasia in infancy (NEHI) (Young et al., 2011), asthma (Sui et al., 2018), and sudden infant death syndrome (SIDS) (Cutz et al., 1996, 2007).

There is accumulated evidence that PNECs represent a heterogenous population of cells. For example, in addition to airway epithelial progenitors that differentiate into PNECs during embryogenesis, basal stem cells (BSCs) have also been shown to generate PNECs (Gu et al., 2014; Montoro et al., 2018). However, it is unclear how these populations differ, whether they are similarly present in mice and humans, and how they originate. Moreover, little is known about the contribution of different populations of PNECs to human diseases,

Here, we identified three distinct types of PNECs in humans and mice that exhibit significant differences in NP expression, homeostatic turnover, and response to injury and disease. We provide evidence that these differences parallel their distinct cell of origin. For clarity, the mouse airway is divided anatomically into the trachea, the main bronchus, and the intrapulmonary airway.

## RESULTS

### TUBB3 analysis reveals three distinct types of PNECs in human airways

**Figure 1.**
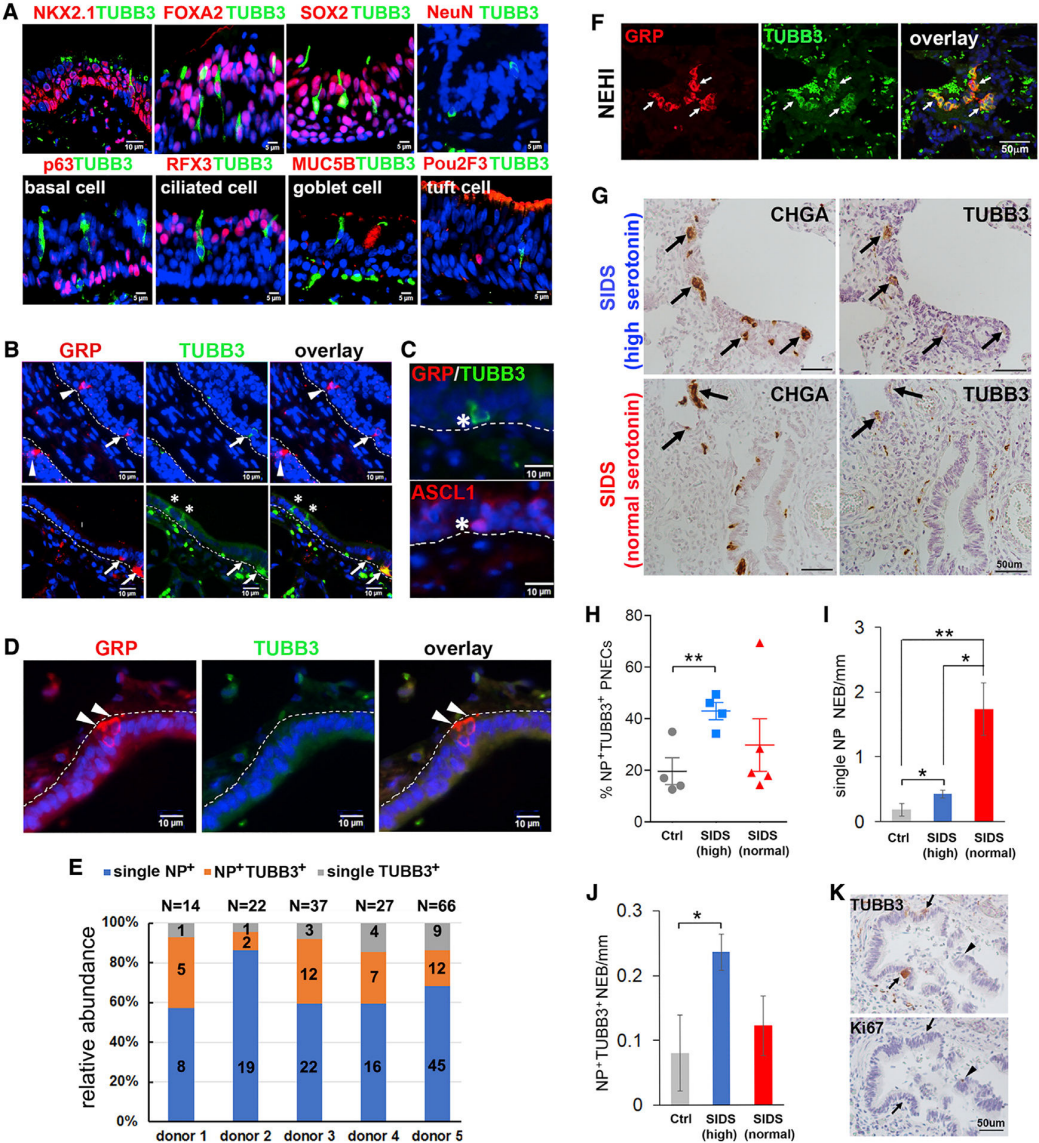
Identification of TUBB3^+^ PNECs in the human lung (A–E) Human airways from healthy donors, sized from 2–3 mm in diameter to bronchioles, were double stained for TUBB3 and specified markers of airway epithelial cells and neurons (NeuNs) in (A), GRP in (B) and (D), and ASCL1 in (C). Arrowhead marks single GRP^+^ PNECs. Arrows mark GRP^+^TUBB3^+^ PNECs. Asterisks mark single TUBB3^+^ PNECs. Images in (C) are from two adjacent sections. The dotted lines in (B)–(D) mark basement membrane beneath the airway epithelium. (E) Quantification of the three types of PNECs in healthy donor lungs. (F) Representative images of double staining for GRP and TUBB3 in a patient with NEHI. Arrows mark GRP^+^TUBB3^+^ PNECs. Background green fluorescence was caused by red blood cells. (G–J) Representative images of CHGA and TUBB3 staining of adjacent sections from SIDS cases (G). Arrows mark CHGA^+^TUBB3^+^ PNECs. Data were quantified as the percentage of PNECs that were NP^+^TUBB3^+^ in (H), the number of single NP^+^ NEBs in (I), and NP^+^TUBB3^+^ NEBs in (J) per millimeter of airway epithelium in non-SIDS controls (n = 4), high 5-HT SIDS cases (n = 4), and normal 5-HT SIDS cases (n = 5). NEBs with more than three PNECs were counted. (K) Representative images of staining for TUBB3 (arrow) and Ki67 (arrowhead) on adjacent sections from SIDS cases. Bar graphs represent means ± SEM. *p < 0.05, **p < 0.01 by Student’s t test. See also Figure S1 and Table S1.

During an analysis of neural innervation of human airways, we discovered rare cells in the airway epithelium expressing TUBB3, a neuron-specific tubulin beta 3 class III (TUBB3) (Figure 1A). Because TUBB3 was not previously known to be expressed by airway epithelial cells, we evaluated these TUBB3^+^ cells by double labeling sections from human donor lungs with a panel of established markers of epithelial cell types. All TUBB3^+^ cells expressed pan-epithelial markers (NKX2.1, FOX2, and SOX2) but not NeuN (neuron), p63 (BSC), RFX3 (ciliated cell), MUC5B (goblet cell), and Pou2F3 (tuft cell) (Figures 1A and S1A). In addition, TUBB3 mostly overlapped with PNEC markers, including GRP, CHGA, synaptic vesicle glycoprotein 2A (SV2), and PGP9.5 (Figures 1B and S1B). To confirm their identity as PNECs, we found that all TUBB3^+^ cells, including a few without GRP expression, were positive for ASCL1, a PNEC-specifying transcriptional factor (Figure 1C) (Borges et al., 1997). Some of the TUBB3^+^ PNECs were in close proximity to nerves (Figure S1C), suggesting that these cells could be innervated. Of note, dendritic cells were shown to express TUBB3 (Lee et al., 2005). However, resident CD11c^+^ dendritic cells in the human airway expressed neither TUBB3 nor GRP (Figure S1D). Lastly, in accord with previous reports (Gosney et al., 1988; Weichselbaum et al., 2005), most PNECs in the human lung were solitary. A few NEBs contained only single GRP^+^ PNECs (Figure 1D), suggesting that TUBB3 may preferentially mark solitary PNECs. From this analysis, we concluded that the human airway contains three types of PNECs: single NP^+^ (the classic PNEC that expresses NP markers but not TUBB3), NP^+^TUBB3^+^ (double positive for both), and single TUBB3^+^ (no NP expression).

We quantified the relative abundance of each PNEC types in healthy human lungs (Table S1). In airways sized from 2 to 3 mm in diameter to bronchioles, all PNECs accounted for 0.3%–0.5% of epithelial cells. Among PNECs, single NP^+^ PNECs were the most abundant (66% ± 12%), followed by NP^+^TUBB3^+^ PNECs (24% ± 11%) and single TUBB3^+^ PNECs (10% ± 4%) (Figure 1E).

### Discrete PNEC types are amplified in different human diseases

The findings above led us to inquire whether diseases associated with neuroendocrine hyperplasia could be distinguished by specific enrichment in any of these PNEC types. For that, we analyzed lung sections from biopsies or autopsies from infants diagnosed with NEHI or SIDS (Table S1). ASCL1 staining of patient samples to identify PNECs proved technically challenging. Because NP (GRP and CHGA) labels approximately 90% of PNECs in the human airway (Figure 1E), NP was used as a marker of PNECs.

NEHI is defined, in part, by an increase in the total number of PNECs, most of which exist as NEBs (Figure 1F) (Young et al., 2011). Immunofluorescence for NP (GRP) and TUBB3 in lung biopsy sections of one NEHI patient (Table S1) revealed many NP^+^TUBB3^+^ NEBs (Figure 1F), increasing the percentage of NP^+^TUBB3^+^ PNECs to 62% compared with 24% ± 11% in healthy lungs (Figure 1E).

Ten SIDS autopsy samples were divided into two groups based on the serum level of serotonin (5-HT) (Haynes et al., 2017). One sample from the high 5-HT group was excluded because of postmortem autolysis (Table S1). There was no difference in postconceptional (PC) age (defined as gestational age plus postnatal age) between the two SIDS groups (high 5-HT, 54.5 ± 8 PC weeks; normal 5-HT, 55.7 ± 20.5 PC weeks). The postmortem interval between death and the start of autopsy was also similar between the two groups (high 5-HT, 19.9 ± 6.7 h; normal 5-HT, 19.67 ± 5.4 h). The percentage of NP^+^TUBB3^+^ PNECs was quantified after single staining of adjacent sections for CHGA and TUBB3 (Figure 1G) and was compared between the two groups of SIDS cases and infants who had died from known causes (Table S1).

Previous reports showed that PNECs are increased in SIDS as are NEB numbers and size (Cutz et al., 1996, 2007). Similarly, we noted readily identifiable NEBs in SIDS lungs (Figure 1G) as compared with the rare NEBs seen in the controls. In the high 5-HT SIDS, the relative abundance of NP^+^TUBB3^+^ PNECs was significantly greater than it was in the control group (42.9% ± 6.6% versus 19.6% ± 10.3%, p < 0.01) (Figures 1G and 1H). In contrast, among the SIDS cases with normal serum levels of 5-HT, except for one outlier, we found no change in the relative abundance of NP^+^TUBB3^+^ PNECs compared with that of controls (Figures 1G and 1H). We also quantified the number of single NP^+^ and NP^+^TUBB3^+^ NEBs relative to airway length in the two SIDS groups. Although all SIDS samples had more single NP^+^ NEBs compared with that of the controls (Figure 1I), only high 5-HT SIDS samples had an increased number of NP^+^TUBB3^+^ NEBs per unit length of airway (Figure 1J).

Taken together, the abundance of NP^+^TUBB3^+^ PNECs is preferentially elevated in high 5-HT SIDS cases, suggesting an increase in NP^+^TUBB3^+^ NEBs as a source of the 5-HT. Lastly, we found no evidence of PNEC proliferation in all SIDS cases, based on a lack of Ki67 labeling (Figure 1K), which indicates that, similar to NEHI (Young et al., 2011), PNEC hyperplasia in SIDS is unlikely to be caused by enhanced PNEC proliferation.

### TUBB3^+^ PNECs are restricted in the mouse trachea and single, NP^+^ PNECs reside in the mouse intrapulmonary airway

**Figure 2.**
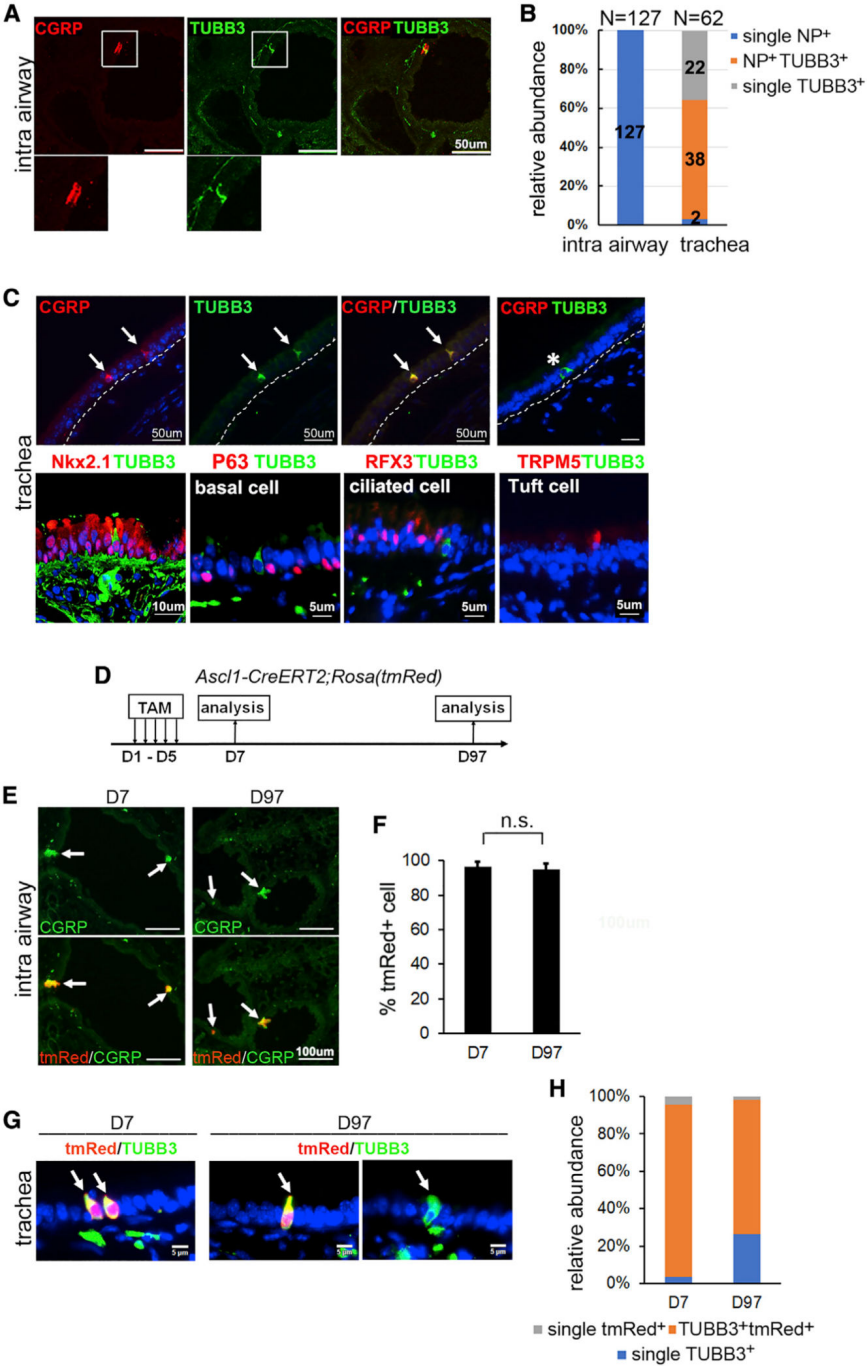
Characterization of the three PNEC types in the trachea and the intrapulmonary airway in mice (A) Representative images of CGRP and TUBB3 staining in the intrapulmonary airway. The outlined area is shown in the bottom panel. n = 6. (B) Relative abundance of the three PNEC types in the intrapulmonary (intra) airway and the trachea. n = 3 mice; five sections from each mouse. (C) Representative images of double staining of mouse tracheal sections for TUBB3 and markers of epithelial cells. Arrows mark CGRP^+^TUBB3^+^ PNECs. Asterisk marks a single TUBB3^+^ PNEC. n = 6. (D) Scheme of neuroendocrine cell lineage tracing using *Ascl1-CreERT2;Rosa(tmRed)* mice. (E–H) Representative images of CGRP staining of the intrapulmonary airway (E) and the trachea (G) in TAM-treated, *Ascl1-CreERT2;Rosa(tmRed)* mice on D7 and D97. Arrow marks tmRed^+^ PNECs. Data were quantified in (F) and (H). n = 3 mice, 3–5 sections from each mouse. See also Figure S2.

We investigated whether these different PNEC types were similarly present in the mouse respiratory tract. In the intrapulmonary airway, independent of the solitary or clustered configuration, PNECs are single NP^+^ type because TUBB3 was found only in nerves associated with NEBs and airway smooth muscle (Figures 2A and 2B). However, recent single-cell RNA analysis identified an extended presence of PNECs in the mouse tracheal epithelium (Montoro et al., 2018). To gain insights into the diversity of these tracheal PNECs, we analyzed the reported single-cell sequencing database. This revealed two PNEC types (NP^+^ or single TUBB3^+^) confirmed by common expression of *Ascl1* but showing clearly distinct global patterns of gene expression (Figures S2). Immunofluorescence for NP (CGRP) and TUBB3 in sections of adult mouse trachea showed that the PNECs were largely NP^+^TUBB3^+^ (61.3%) and single TUBB3^+^ cells (35.5%), with only a few single NP^+^ PNECs (3.2%) (Figures 2B and 2C). TUBB3^+^ cells were also found in main bronchi in mice but at too few to allow quantification.

To test whether the TUBB3^+^ cells (especially the single TUBB3^+^ PNECs) in the mouse trachea originated from a common Ascl1^+^ progenitor, we performed a lineage tracing assay in *Ascl1-CreERT2;Rosa(tmRed)* mice and assessed tmRed colocalization with CGRP and TUBB3 at day 2 (D7) and at 3 months (D97) post-tamoxifen (post-TAM) injection (Figure 2D). In intrapulmonary airways, almost all CGRP^+^ PNECs (96.7% ± 3.9%) were tmRed^+^ (Figure 2E) at D7, confirming the high efficiency and stringency of lineage tracing. There was no change in the high percentage of tmRed^+^ PNECs at D97 compared with that of D7 (Figures 2E and 2F). Therefore, single NP^+^ PNECs in the mouse intrapulmonary airway are either long lived or are maintained by self-renewal. In the trachea of TAM-treated, *Ascl1-CreERT2;Rosa(tmRed)* mice, 95.3% of tmRed^+^ PNECs (101/106) were TUBB3^+^ at D7 (Figures 2G and 2H). A few tmRed^+^TUBB3^−^ cells may be undifferentiated PNEC progenitors. In addition, 96.2% of TUBB3^+^ cells were tmRed^+^ (Figure 2H). At D97, the percentage of TUBB3^+^ tmRed^+^ cells (85/116) was reduced by ~20% compared with that of D7 (Figures 2G and 2H), consistent with homeostatic replenishment of tmRed^+^ PNECs by an unlabeled progenitor population (BSCs) (Montoro et al., 2018). However, similar to the results from D7, almost all tmRed^+^ cells remained TUBB3^+^ at D97 (85/87) (Figure 2H). These findings indicate that TUBB3 is a persistent marker of PNECs in the mouse trachea.

### BSCs generate TUBB3^+^ PNECs

**Figure 3.**
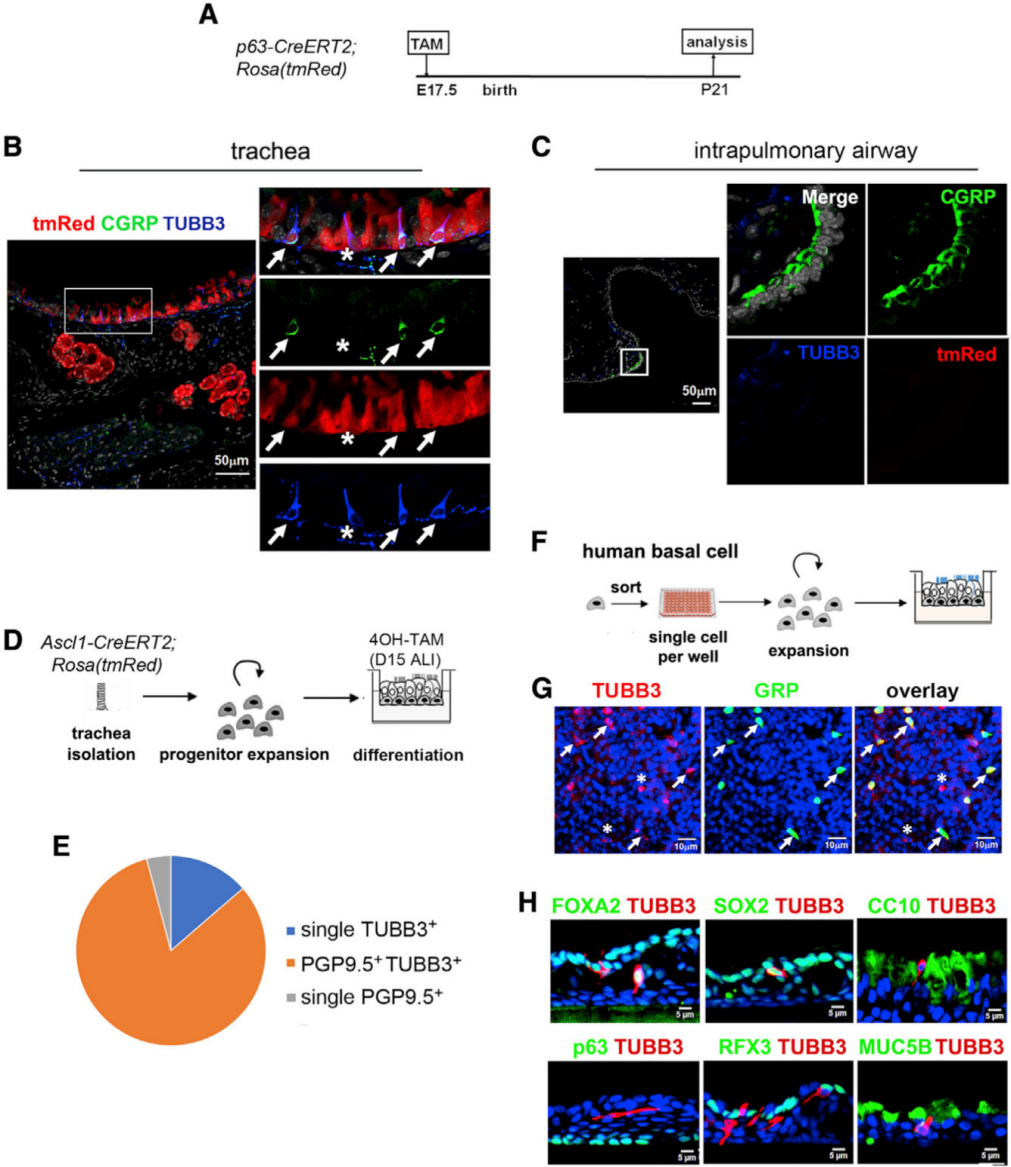
BSCs generate TUBB3^+^ PNECs in culture (A) Scheme of BSC lineage tracing in TAM-inducible, *p63-CreERT2;Rosa(tmRed)* mice. (B and C) The trachea (B) and the intrapulmonary airway (C) were analyzed using 6–12 fields (20×) per sample and a total of three mice. See also Figures S3 and S4. (D) Scheme of PNEC lineage labeling of the *Ascl1-CreERT2;Rosa(tmRed)* BSC culture. The culture was treated with 4-OH-TAM on day 15 ALI and analyzed on days 17–19 of ALI. (E) Relative abundance of the three PNEC types in ALI culture. (F) Scheme of human BSC culture in ALI. (G and H) Day 14 ALI culture was analyzed by staining for TUBB3 and the specified markers. In (B) and (G), arrows show GRP^+^TUBB3^+^ PNECs, and asterisks mark single TUBB3^+^ cells. Data are representative results using BSCs from three donors.

To investigate whether tracheal TUBB3^+^ PNECs originate from BSCs, we performed BSC lineage tracing in *p63-CreERT2;Rosa(tmRed)* mice. We have previously shown that TAM exposure at embryonic day (E) 17.5 results in robust (~90%) BSC labeling of the tracheal epithelium of 3-week-old pups (Yang et al., 2018). We found tmRed^+^ tracheal labeling in both NP^+^TUBB3^+^ and single TUBB3^+^ PNECs (Figures 3A and 3B). No tmRed^+^ cells were found in the intrapulmonary airway (Figure 3C). Similar results were obtained after BSC lineage labeling in adult mice (Figures S3A and S3B). Therefore, mouse BSCs generate TUBB3^+^ PNECs in the trachea but not elsewhere, consistent with the restricted BSC distribution in extrapulmonary airways.

Given that the airway repair after H1N1 influenza viral infection included the expansion of basal-like (p63^+^ and Krt5^+^) endogenous progenitors (Zuo et al., 2015; Yang et al., 2018), we asked whether this involved induction of any of the PNEC types. We performed a lineage-tracing analysis of *p63-CreERT2;Rosa(tmRed)* adult mice before H1N1 viral infection. Staining of the intrapulmonary airway at 15 day after infection showed no p63 lineage labeling in CGRP^+^ PNECs that remained single NP^+^ (Figure S3C). In addition, club cells could not give rise to single NP^+^ PNECs in the intrapulmonary airway at baseline and in injury repair by lineage-tracing analysis using H1N1-infected, *CC10-CreERT2;Rosa(tmRed)* mice (Figures S3D–S3F). Furthermore, we found no evidence of proliferation of single NP^+^ PNECs during the course of H1N1-induced injury repair, despite robust Ki67 staining in other airway epithelial cell types (Figure S4). Thus, BSCs and club cells cannot generate single NP^+^ PNECs in the mouse intrapulmonary airway under H1N1-injury conditions.

We further investigated BSC as the progenitors of TUBB3^+^ PNECs by isolating and expanding tracheal BSCs from adult Ascl1-*CreERT2;Rosa(tmRed)* mice. These cells were cultured at the air-liquid interface (ALI) to induce differentiation and were treated with 4-hydroxytamoxifen (4-OH-TAM) at ALI day 15 (Figure 3D). Analysis of these cultures 2–4 days later showed almost all tmRed^+^ cells (99.3%) were single or double positive for PGP9.5 and TUBB3. Most TUBB3^+^ cells expressed PGP9.5 (518/604) (Figure 3E). Therefore, lineage labeling both *in vivo* and *in vitro* consistently shows that BSCs generate almost exclusively TUBB3^+^ PNECs.

To test whether BSCs were also the progenitor of TUBB3^+^ PNECs in human airways, we isolated bronchial BSCs from healthy adult lung donors (Mou et al., 2016). To eliminate possible contamination from neurons, we sorted epithelial cell adhesion molecule positive (EpCAM^+^) cells and seeded the cells at one cell per well (Figure 3F). At ALI day 14, TUBB3 immunoreactivity was identified in 0.2%–0.5% of the cells (Figure 3G); 87% of the TUBB3^+^ cells were GRP^+^, and 98% of the GRP^+^ cells expressed TUBB3 (Figure 3G). Furthermore, all TUBB3^+^ cells expressed pan-epithelial markers (SOX2 and FOXA2) but lacked the markers for club cells, basal cells, ciliated cells, and goblet cells (Figure 3H). These findings indicate that human BSCs differentiate into NP^+^TUBB3^+^ and single TUBB3^+^ PNECs.

### TUBB3 is required for cellular protrusions of PNECs

**Figure 4.**
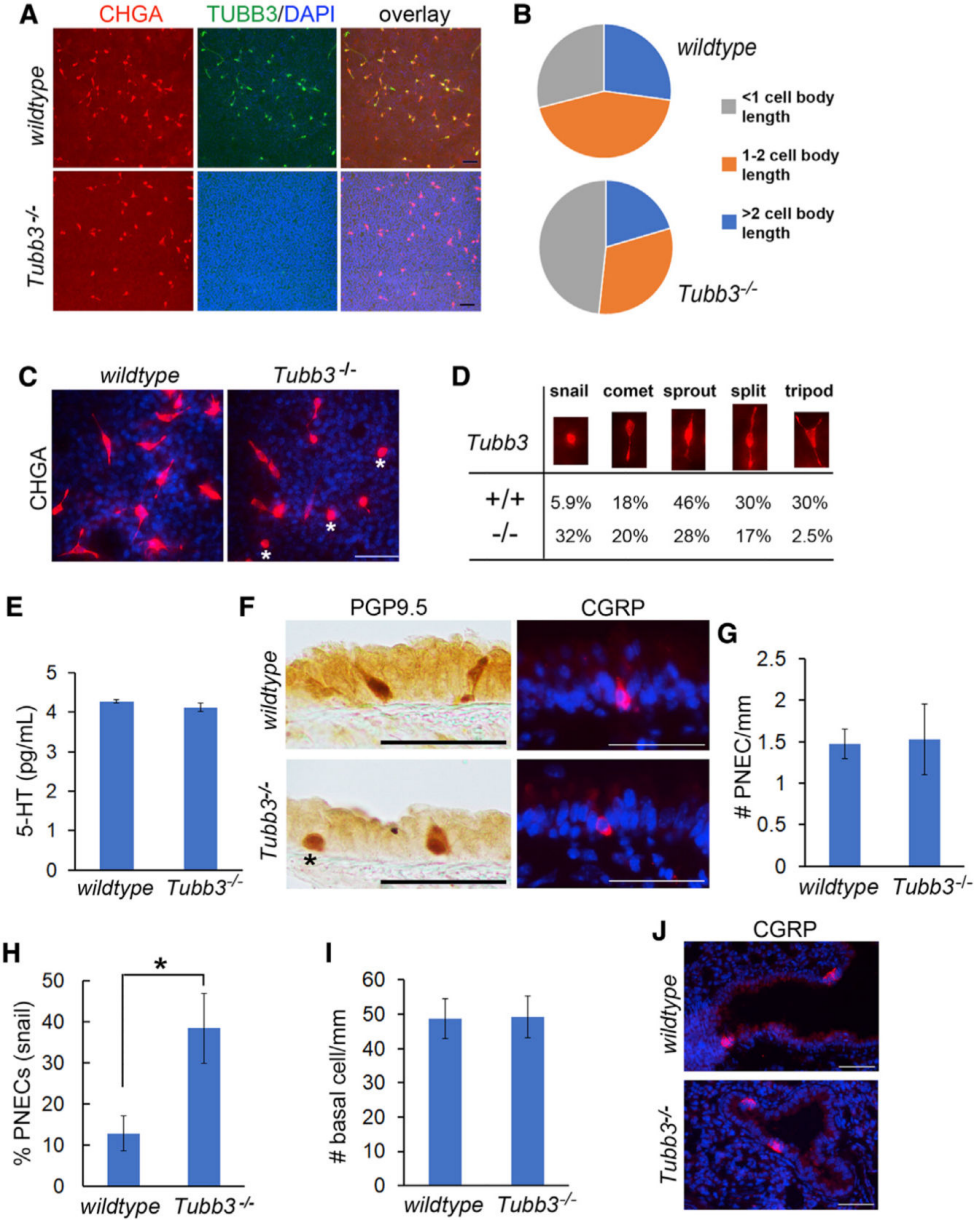
TUBB3 is required for PNECs to form cellular protrusions (A and C) Representative images of double staining for CHGA and TUBB3 in ALI culture of wild-type and *Tubb3*^*−/−*^ BSCs. Asterisks mark “snail” PNECs with very short protrusions. (B and D) Relative abundance of CHGA^+^ PNECs with protrusions of different lengths (B) and patterns (D) in wild-type and *Tubb3*^*−/−*^ cultures. More than 500 PNECs were counted in each experiment, with a total of two independent experiments. (E) 5-HT secretion in ALI culture of wild-type and *Tubb3*^*−/−*^ BSCs by ELISA. Cultures in triplicates and from two independent experiments were assayed. (F) Representative images of PNECs in the trachea of wild-type and *Tubb3*^*−/−*^ mice. An asterisk marks a snail PNEC. (G–I) Quantification of PGP9.5^+^ PNECs in (G), snail PNECs in (H), and p63^+^ BSCs in (I) per millimeter along the tracheal epithelium in wild-type and *Tubb3*^*−/−*^ mice. (J) Representative images of CGRP^+^ NEBs in the intrapulmonary airway of wild-type and *Tubb3*^*−/−*^ mice. n = 15 sections from three mice of each genotype. Bar graphs represent means ± SEM. *p < 0.05 by Student’s t test. Scale bars, 50 μm.

TUBB3 is a constituent of microtubules in neurons that act in concert with neurofilaments to maintain cell morphology and transport organelles and synaptic vesicles for communication with other neurons and innervated peripheral tissue (Prokop, 2020). *Tubb3*^*−/−*^ mice are normal under homeostasis but exhibit delayed axon regeneration after nerve injury (Latremoliere et al., 2018). The role of TUBB3 in the airway epithelium has not been studied. To address that issue, we isolated basal cells from wild-type and *Tubb3*^*−/−*^ adult tracheas and induced differentiation in ALI cultures. We found that TUBB3 deficiency had no effect on PNEC differentiation, NP expression, or baseline secretion of 5-HT (Figures 4A and 4E). Staining showed that wild-type PNECs displayed several patterns of cellular protrusions: “snail” (very short protrusion), “comet” and “sprout” (one protrusion), “split” (two protrusions), and “tripod” (3 protrusions) (Figures 4B–4D and S1F). In contrast, *Tubb3*^*−/−*^ PNECs had significantly shorter protrusions, causing an increase in the abundance of snail PNECs and reciprocal decreases in split and tripod PNECs (Figures 4A–4D).

We then assessed the phenotype of PNECs *in vivo* in the trachea of *Tubb3*^*−/−*^ adult mice. We found most (85%) of the PNECs in the wild-type trachea extended protrusions (Figures 2C, 2G, 3B, 4F, and 4H). Similar protrusions of PNECs were also found in the human airway (Figures 1A, 1B, and S1) (Weichselbaum et al., 2005). In comparison, more than 40% of PNECs lacked apical protrusions in the trachea of *Tubb3*^*−/−*^ mice (Figures 4F and 4H). However, TUBB3 deficiency had no effect on basal-lateral distribution of CGRP in PNECs (Figure 4F), the number of PNECs and BSCs in the trachea (Figures 4G and 4I), and the abundance of NEBs in the intrapulmonary airway that normally lack TUBB3 expression (Figure 4J). Therefore, TUBB3 is critical for apical and basal protrusions of PNECs.

## DISCUSSION

Here, we identified three distinct types of PNECs occurring in the airways of mice and humans, and we provide insights into their ontogeny, phenotype, and occurrence in health and disease. The hierarchical relationship between the two new TUBB3^+^ PNEC types (NP^+^TUBB3^+^ and single TUBB3^+^ PNECs) warrant future investigation. Mechanisms that regulate BSC differentiation into TUBB3^+^ PNECs are also unknown. A recent study has shown that BSCs directly differentiate into PNECs under hypoxia (Shivaraju et al., 2021), which may explain the hyperplasia of NP^+^TUBB3^+^ PNECs in NEHI and a subset of SIDS cases without the involvement of PNEC proliferation.

Cellular protrusions mediate the communication between the cell and the environment. We show that TUBB3^+^ PNECs are dependent on TUBB3 to extend apical and basal protrusions. As such, TUBB3 may endow PNECs with unique apical-to-basal communication, such that changes in the airway lumen can be sensed by the apical protrusion and transmitted to neighboring cells via NP and amines released from the basal protrusion. In contrast, NEBs do not express TUBB3 and appear to lack similar protrusions as TUBB3^+^ PNECs. We, thus, speculate that TUBB3^+^ PNECs and TUBB3^−^ NEBs may have different sensing mechanisms.

We noted in SIDS that, compared with controls, cases with normal 5-HT levels had an increase in NP^+^ NEBs, whereas high 5-HT cases showed an increased number of NP^+^TUBB3^+^ NEBs. Given that these cells have been shown to serve as O_2_/CO_2_/pH sensors (Domnik and Cutz, 2011), an increase of NEBs in SIDS suggests changes in the lungs of SIDS infants in response to respiratory challenges. Novel to this study is the identification of the TUBB3^+^ PNECs in SIDS and their association with increased levels of serum 5-HT. It has been postulated that NEBs are a source of the increased serum 5-HT reported in a subset of SIDS infants (Haynes et al., 2017).

The present study generates new hypotheses about 5-HT-specific pathways of TUBB3^+^ NEBs in SIDS infants, especially given that TUBB3 in the mouse is not required for 5-HT secretion. Associations between peripheral serotonergic abnormalities, including those of pulmonary origin, and central serotonergic abnormalities (Duncan et al., 2010) are of interest to better understand the different etiologies of SIDS pathogenesis and the potential role of TUBB3^+^ PNEC-derived 5-HT in this etiology. In regard to NEHI, it is unknown whether patients with NEHI have high levels of blood or serum 5-HT.

Our findings demonstrate significant differences between humans and mice with respect to the distribution and clustering of PNECs. For human airway diseases that involve the hyperplasia of BSC-derived, TUBB3^+^ PNECs, such as NEHI and SIDS, the mouse intrapulmonary airway may be a limited model for mechanistic studies. Experimental systems derived from the mouse tracheal explant and airway BSCs may serve as a more disease-relevant model.

## STAR★METHODS

### RESOURCE AVAILABILITY

#### Lead contact

Further information and requests for resources and reagents should be directed to and will be fulfilled by the lead contact, Xingbin Ai (xai@mgh.harvard.edu).

#### Materials availability

This study did not generate new unique reagents.

#### Data and code availability

This study did not generate any unique datasets or code.

### EXPERIMENTAL MODELS AND SUBJECT DETAILS

#### Mice

*p63-CreERT2* mice were generated and characterized as described previously (Lee et al., 2014). The *Rosa-tmRed* line (stock number: 007914), *CC10-CreERT2* line (stock number: 016225), *Ascl1-CreERT2* line (stock number: 012882) were purchased from The Jackson Laboratory. *Tubb3*^*−/−*^ mouse line was kindly provided by Dr. Engle at Boston Children’s Hospital, Harvard Medical School and was characterized previously (Latremoliere et al., 2018). For time-pregnancy experiments, male mice carrying the *CreERT2* allele were mated with *Rosa-tmRed* female mice. Noon of the day when the vaginal plug was identified was determined as gestation day 0.5 (E0.5). The stages of embryogenesis for linage tracing was E13.5 and E17.5. All other experiments used adult mice at minimal 8 weeks of age. All studies were approved by Institutional Animal Care and Use committees at Columbia University and Massachusetts General Hospital, Harvard Medical School.

#### Human donor lungs

Lungs from de-identified, male and female donors between 0–65 years of age were purchased from International Institute for the Advancement of Medicine (IIAM), a non-profit institute that provides non-transplantable human tissues for medical research. 6 human donor lungs between 0–65 years of age (4 males and 2 females) were used in this study. These donors had no previous lung diseases, and mostly died of brain injuries and heart failure. All the information regarding the donor is anonymous. Because the experiment involves no intervention or interaction with the donors and relates to no living individuals, this project is deemed non-human subject research by institutional IRB committee.

#### Histological sections of lung biopsy samples from NEHI and SIDS patients

The tissue sections of the NEHI patient were provided by Boston Children’s Hospital (BCH). The tissue sections of SIDS cases, and 3 of the 4 non-SIDS control cases were provided by the San Diego Office of the Medical Examiner (SDME), San Diego, CA, which is available to us for research under the auspices of California Law, Chapter 955, Statutes of 1989. Serum serotonin values from the SIDS cases and SDME controls were previously reported by Haynes et al. (2017) (Table S1). This study was approved by IRB at Boston Children’s Hospital.

#### Primary human airway basal cells

Human adult airway basal cells were isolated from fresh discarded lung tissues from surgical specimens in MGH under an IRB-approved protocol (#2017P001479, Dr. Hongmei Mou) through lung transplantation team in MGH.

### METHOD DETAILS

#### Lineage tracing analysis

Lineage tracing experiments were performed after mating male *CreERT2* mice with *Rosa(tmRed)* female mice. Tamoxifen (TAM) was dissolved in sunflower seed oil at a concentration of 10 mg/mL (Sigma, T5648). For lineage tracing of basal stem cells in embryos, TAM was administered by gavage to pregnant mice at gestation day 13.5 and E17.5 (160 μg/g body weight) (Yang et al., 2018). For lineage tracing in postnatal mice, TAM (240 μg/g body weight) was given by oral gavage to both male and female mice of 3–5 weeks of age. The trachea and the intrapulmonary airway samples from *Ascl1-CreERT2;Rosa(tmRed)* mice were harvested 2 days and 3 months after the last TAM injection. The tissue samples from *p63-CreERT2;Rosa(tmRed)* mice and *CC10-CreERT2;Rosa(tmRed)* mice were previously described (Yang et al., 2018). Samples were analyzed for CGRP and TUBB3 expression and colocalization with tmRed.

#### H1N1 (PR8) viral preparation and infection

The lung samples following H1N1 infection were previously described (Yang et al., 2018) and were re-analyzed in this study for CGRP and TUBB3 expression. Briefly, adult mice were anesthetized by isoflurane followed intranasal administration of 120pfu of H1N1 virus in 30μl PBS. Control mice received PBS. Lungs were inflated with 4% paraformaldehyde in PBS and fixed overnight before samples were processed for paraffin-embedding. Tissue sections (6–8μm) were rehydrated before citric acid-based antigen retrieval and antibody staining following established protocols (Yang et al., 2018).

#### Airway basal cell culture

Human and mouse basal cells were isolated and cultured using the established protocols (Mou et al., 2016; Levardon et al., 2018). Briefly, basal cells were maintained in Small Airway Epithelial Cell Medium (Lonza, CC-3118 or Promocell medium, C-21170) with 5–10 μM ROCKi (Tocris, 1254), 1 μM A-83–01 (Tocris, 2939), 0.1 μM DMH-1 (Tocris, 4126) and 0.5 μM CHIR99021 (Tocris, 4423). Human cells were cultured on plates pre-coated with laminin-enriched 804G-conditioned medium, while mouse cells were cultured on plain plates without pre-coating. To expand, cells were dissociated with trypsin and re-seeded at 1:10 ratio. Mucociliary differentiation of airway basal cells in ALI was performed as previously described (Mou et al., 2016). Briefly, airway basal cells were seeded onto 0.4 μm transwell membranes pre-coated with 804G-conditioned medium at a cell density of > 6000 cells/mm^2^ and were allowed to attach for a minimum of 12 hours. After the removal of unattached cells, the medium was replaced with complete Pneumacult-ALI medium (StemCell Technology, Cat. 05001) filling both the upper and lower chambers. The next day, the ALI medium was added only to the lower chamber to initiate differentiation at the airway-liquid interface. The ALI medium was changed daily until differentiation was well established after 14 days as a standard protocol. To lineage label PNECs in ALI culture derived from *Ascl1-CreERT2;Rosa(tmRed) mice*, 4-OH-tamoxifen (1 μM, Sigma-Aldrich, H7904) was added to day 15 ALI culture. After 24 hours, the culture was rinsed with saline three times to remove 4-OH-tamoxifen. Cultures were fixed for staining on day 17–19 in ALI. For the measurement of serotonin secretion, ALI culture was replaced with fresh media and after 2 days, media were collected for ELISA for 5-HT using a commercialized kit.

#### Antibody staining

Protocols of antibody staining were previously published (Mou et al., 2016; Barrios et al., 2017). Briefly, for staining of the ALI culture, the transwell membrane was fixed with 4% PFA at room temperature for 10–15 min, followed by washing and permeabilization with PBS + 0.2% Triton X-100. The membrane was either used for wholemount staining or embedded in optimum cutting temperature compound for 4–7 μm frozen sections. For staining of paraffin sections, sections were re-hydrated before incubating with primary antibodies. For mouse monoclonal antibodies, M.O.M kit (Vector Labs BMK-2202) was used. Sections were stained using a standard staining protocol (Mou et al., 2016; Barrios et al., 2017). Immunofluorescence of cells on transwell membranes was visualized with the Olympus IX81 inverted fluorescence microscope. Images are captured at multiple focal planes and combined using MicroSuite FIVE (Olympus Soft Imaging Solutions) and Extended Focal Imaging (EFI) module to create a single in-focus image, capturing the cellular complexities of the thicker ALI cultures. Bright field images were taken using a digital camera (Nikon DS-Fi2). Primary antibodies include: rabbit anti-GRP (1:200, Immunostar, Cat#20073), rabbit anti-Chromogranin A (1:200 Abcam, ab45179), goat anti-CC10 (1:500, Santa Cruz, Cat#SC-9772), mouse anti-TUBB3 (TuJ1) (1:50, R&D Systems, MAB1195), rabbit anti-Nkx2.1 (1:100, Abcam, ab76013), rabbit anti-CGRP (1:1000, Sigma-Aldrich, C8198), goat anti-Foxa2 (1:200, Santa Cruz technology, sc-655), rabbit anti-NeuN (1:300, Abcam, Cat#ab190566), rabbit anti-p63 (1:200, Genetex, GTX102425), goat anti-Sox2 (1:200, R&D systems, AF2018), rabbit anti-RFX3 (1:500, Sigma, HPA035689), rabbit anti-Pou2F3 (1:250, Sigma-Aldrich, HPA019652), mouse anti-SV2 (1;50, Developmental Studies Hybridoma Bank), mouse anti-Ezrin (1:50, Developmental Studies Hybridoma Bank), rabbit anti-Ki67 (1:300, Cell Marque, Cat#SP6), mouse anti-Muc5B (1:200, Sigma-Aldrich, HPA008246). The following secondary antibodies were used: biotinylated goat anti-rabbit (1:200, Vector Laboratories, BA-1000), donkey anti-rabbit (conjugated with Alexa Fluor 647), donkey anti-mouse (conjugated with Alexa Fluor 488), donkey anti-rat (conjugated with Alexa Fluor 647). All conjugated secondary antibodies were purchased from Thermo Fisher Scientific. Nuclei were counterstained by DAPI.

### QUANTIFICATION AND STATISTICAL ANALYSIS

#### Quantification of the abundance of 3 PNEC types in human lung tissue

For quantification of PNECs in healthy donor lungs, the number of fluorescently labeled cells that were single and doubly positive for GRP and TUBB3 were counted from the entire tissue section following double staining. For each donor, 3 sections from different biopsy samples were quantified and added together to represent one donor. For quantification of PNECs in the NEHI sample, the airways in the entire section, a total of 2 non-overlapping sections, were quantified for fluorescently positive cells after double staining for GRP and TUBB3. For quantification of PNECs in SIDS biopsy samples, adjacent tissue sections were single labeled for CHGA and TUBB3, respectively. Labeled cells were counted throughout the sections via a Neurolucida cell counting program (MBF Bioscience, Williston, VT). The percentage of a specific PNEC subpopulation was quantified by dividing the number of the specific PNECs by the total number of PNECs. For quantification of NEBs in SIDS cases, NEBs with 3 or more PNECs were counted on the entire tissue section. The length of the airway was measured by NIH ImageJ. For quantification of the proliferation of PNECs in SCID cases, adjacent tissue sections were single labeled for TUBB3 and Ki67, respectively.

#### Quantification of the 3 PNEC types in the trachea and the intrapulmonary airway in mice

For quantification of PNECs in mouse trachea, 5 longitudinal sections were used for each sample. For quantification of solitary PNECs and NEBs in mouse intrapulmonary airway, at least 3 sections were stained and 6–12 non-overlapping images (20X) images were quantified for each sample. Results represent the quantification from 3 mice in each condition. The length of tracheal airway epithelium was measured by NIH ImageJ.

#### Quantification of PNECs in ALI culture

Whole mount staining of the transmembrane was performed before the membrane was imaged. PNECs were counted from 5 non overlapping images. The total number of cells were measured based on DAPI labeling. Results represent the quantification from a minimum of 2 independent experiments.

#### Statistical analyses

Quantification and statistical analyses were described in the Method details section above and Figure legends associated with each experiment. GraphPad Prism 6 was used for data analysis. Data in all bar graphs represent mean ± SEM from at least 2 independent experiments. For comparisons between two conditions, statistical significance was analyzed using the unpaired Student’s t test. The difference between experimental groups was considered statistically significant if *P value* was less than 0.05.

## Supplementary Material

1

## Figures and Tables

**KEY RESOURCES TABLE:** 

REAGENT or RESOURCE	SOURCE	IDENTIFIER
Antibodies		
rabbit polyclonal anti-GRP	ImmunoStar	Cat#20073; RRID: AB_572221
rabbit polyclonal anti-p63	Genetex	Cat# GTX102425, RRID:AB_1952344
rabbit polyclonal anti-CGRP	Sigma-Aldrich	Cat#C8198; RID:AB_259091
rabbit polyclonal anti-Nkx2.1	Abcam	Cat#ab76013; RRID:AB_1310784
goat anti-CC10	Santa Cruz	Cat#sc-9772; RRID:AB_2238819
goat anti-Sox2	R&D systems	Cat# AF2018, RRID:AB_355110)
goat anti-Foxa2	R&D systems	Cat# AF2400, RRID:AB_2294104
rabbit polyclonal anti-RFX3	Sigma	Cat# HPA035689, RRID:AB_10671224
rabbit polyclonal anti-Pou2f3	Sigma-Aldrich	Cat# HPA019652, RRID:AB_185558
hamster monoclonal anti-CD11c, APC, clone N418	eBioscience	Cat#17–0114-81; RRID: AB_469345
mouse monoclonal anti-Ascl1	BD Biosciences	Cat# 556604, RRID:AB_396479
rabbit polyclonal anti-Muc5b	Sigma-Aldrich	Cat# HPA008246, RRID:AB_1854203
mouse monoclonal anti-SV2	Developmental Studies Hybridoma Bank	N/A
mouse monoclonal anti-neural class III beta-tubulin, Clone Tuj1	R&D Systems	Cat#MAB1195; RRID: AB_357520
Rabbit monoclonal anti-Ki67	Cell Marque	Cat# SP6
rabbit anti-CHGA	Abcam	Cat# ab45179, RRID:AB_726879
rabbit polyclonal anti-NeuN	Abcam	Cat#ab190565, RRID:AB_2732785
chicken polyclonal anti-Krt5	BioLegend	Cat#905901, RRID:AB_2565054
APC anti-human CD326 (EPCAM)	BioLegend	Cat# 324207, RRID:AB_756081
donkey anti-goat; Alexa Fluor 488	Thermo Fisher	Cat# A-11055; RRID:AB_2534102
donkey anti-goat; Alexa Fluor 568	Thermo Fisher	Cat# A-11057; RRID:AB_2534104
donkey anti-rabbit; Alexa Fluor 568	Thermo Fisher	Cat#A10042; RRID:AB_2534017
donkey anti-rabbit; Alexa Fluor 488	Thermo Fisher	Cat# A-21206;RRID:AB_2535792
donkey anti-rabbit; Alexa Fluor 647	Thermo Fisher	Cat# A-31573; RRID:AB_2536183
donkey anti-rat; Alexa Fluor 488	Jackson ImmunoResearch	Cat#712–546-153; RRID:AB_2340686
donkey anti-mouse; Alexa Fluor 488	Thermo Fisher	Cat# A-21202; RRID:AB_141607
donkey anti-mouse; Alexa Fluor 568	Thermo Fisher	Cat# A10037; RRID:AB_2534013
biotinylated polyclonal anti-rabbit	Vector Laboratories	Cat# BA-1000, RRID:AB_2336201
Biotinylated polyclonal anti-mouse	Vector Laboratories	Cat# BA-2000, RRID:AB_2313581
Biological samples		
human lungs from donors with no previous airway diseases	International Institute for the Advancement of Medicine	https://iiam.org/
tissue sections of a NEHI case	Boston Children’s Hospital (BCH)	N/A
tissue sections of non-SIDS controls and SIDS cases	San Diego Medical Examiner (SDME)	N/A
Chemicals, peptides, and recombinant proteins		
Tamoxifen	Sigma-Aldrich	Cat#T5648
4-hydroxy-tamoxifen	Sigma-Aldrich	Cat#H7904
Sunflower seed oil	Sigma-Aldrich	Cat#S5007
Y-27632 dihydrochloride	Tocris	Cat#1254
A-83–01	Tocris	Cat#2939/10
DMH-1	Tocris	Cat#4126
CHIR99021	Tocris	Cat#4423
Critical commercial assays		
Mouse on Mouse Kit	Vector Laboratories	Cat#BMK-2202
Standard ABC kit	Vector Laboratories	Cat#PK-6100
DAB Peroxidase Substrate Kit	Vector Laboratories	Cat# SK-4100
Serotonin ELISA Kit	Enzo Life Sciences	Cat# AD1–900-175
Experimental models: Organisms/strains		
Mouse: *Rosa(tmRed)*	The Jackson Laboratory	JAX: 007914
Mouse: *Ascl1-CreERT2*	The Jackson Laboratory	JAX: 012882
Mouse: *CC10-CreERT2*	The Jackson Laboratory	JAX: 016225
Mouse: *p63-CreERT2*	Lee et al., 2014	N/A
Mouse: *TUBB3*^*−/−*^	Latremoliere et al., 2018	N/A
Software and algorithms		
GraphPad Prism 6	GraphPad Software	https://www.graphpad.com
ImageJ 1.49v	ImageJ	https://imagej.nih.gov

## Highlights

- Rare TUBB3^+^ solitary pulmonary neuroendocrine cells reside in the conducting airway
- Airway basal stem cells generate TUBB3^+^ pulmonary neuroendocrine cells
- TUBB3 is required for cellular protrusions of pulmonary neuroendocrine cells
- TUBB3^+^ pulmonary neuroendocrine cells are amplified in infant diseases
