# Supplementary material for: Airway basal stem cells generate distinct subpopulations of PNECs
Source: Cell Rep. Author manuscript; Available in PMC 2021 May 22. (PMC8140387; doi:10.1016/j.celrep.2021.109011)
Supplement: 1 [file NIHMS1696730-supplement-1.pdf]

Cell Reports, Volume *35*

Supplemental information

Airway basal stem cells generate distinct subpopulations of PNECs

Hongmei Mou, Ying Yang, Molly A. Riehs, Juliana Barrios, Manjunatha Shivaraju, Adam L. Haber, Daniel T. Montoro, Kimberly Gilmore, Elisabeth A. Haas, Brankica Paunovic, Jayaraj Rajagopal, Sara O. Vargas, Robin L. Haynes, Alan Fine, Wellington V. Cardoso, and Xingbin Ai

Supplementary document

Supplementary Figures 1-4

Supplementary Table 1: Database of human tissue; cases and controls.

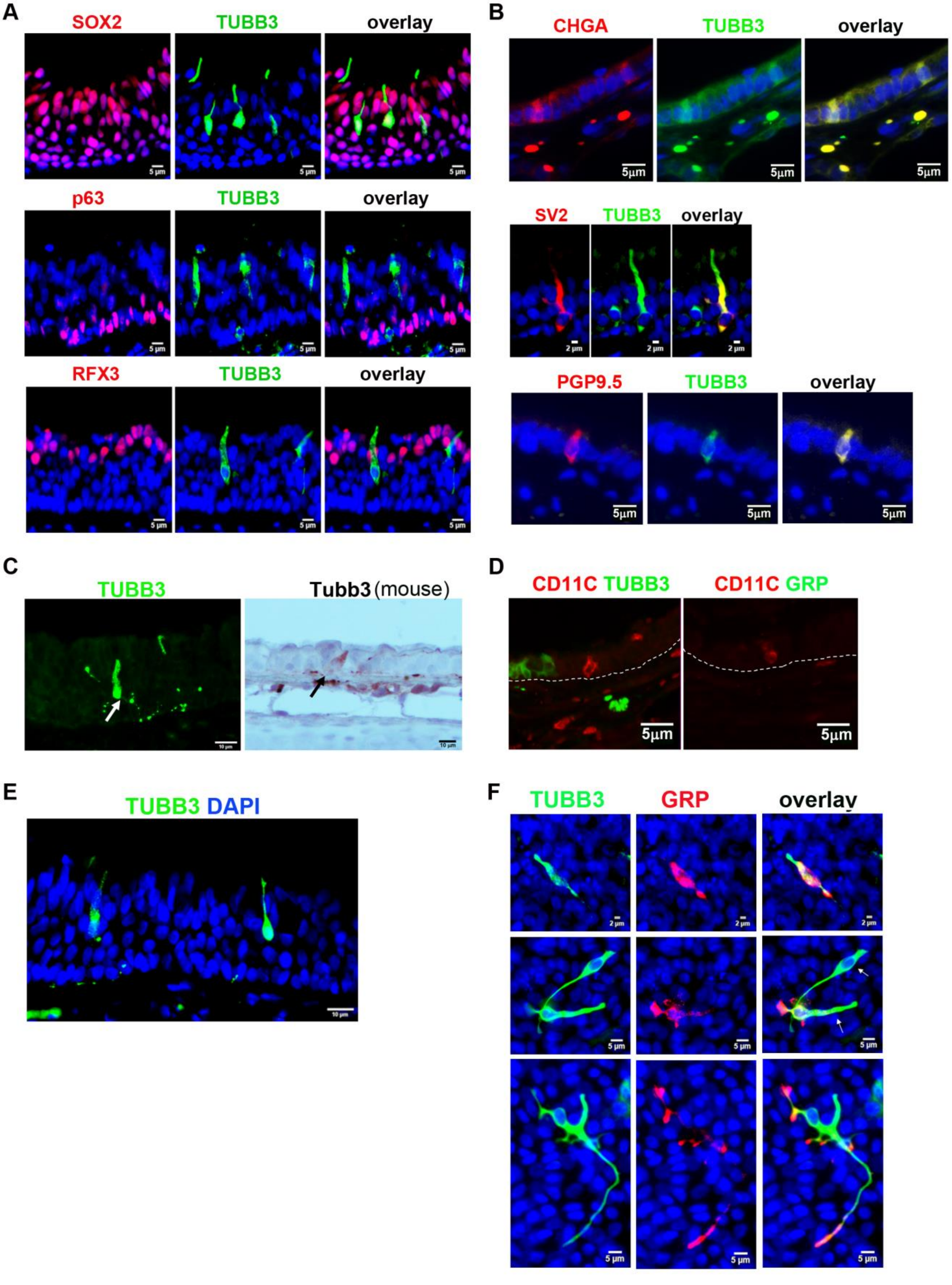
A
SOX2
TUBB3
overlay
p63
TUBB3
overlay
RFX3
TUBB3
overlay
B
CHGA
TUBB3
overlay
5µm
SV2
TUBB3
overlay
2 µm
PGP9.5
TUBB3
overlay
5µm
C
TUBB3
Tubb3 (mouse)
D
CD11C TUBB3
CD11C GRP
5µm
E
TUBB3 DAPI
F
TUBB3
GRP
overlay
2 µm
5 µm

Figure S1. TUBB3 identifies PNECs in the human airway and the mouse trachea. Related to Figure 1 and Figure 4. (**A**) Representative single staining images for selected, double stained panels of the human airway in Figure 1A. (**B**) Representative images of double staining for TUBB3 and synaptic vesicle glycoprotein 2A (SV2), CHGA, and PGP9.5 in human lung sections. Nuclei were counterstained with DAPI. (**C**) TUBB3 staining of the human airway and the mouse trachea. Arrow marks TUBB3$^+$ nerves in the proximity of a TUBB3$^+$ PNEC. (**D**) Representative images of double staining of human lung tissue sections for CD11c and TUBB3 and CD11C and GRP. The dotted line marks the basement membrane beneath the airway epithelium. (**E**) Images of TUBB3$^+$ PNECs with cellular protrusions that extend into the apical surface of the human airway epithelium. (**F**) Double staining of the ALI culture of human BSCs for TUBB3 and GRP. Images show various patterns of cellular protrusions of human PNECs in culture.

Figure S2_Mou, et al.

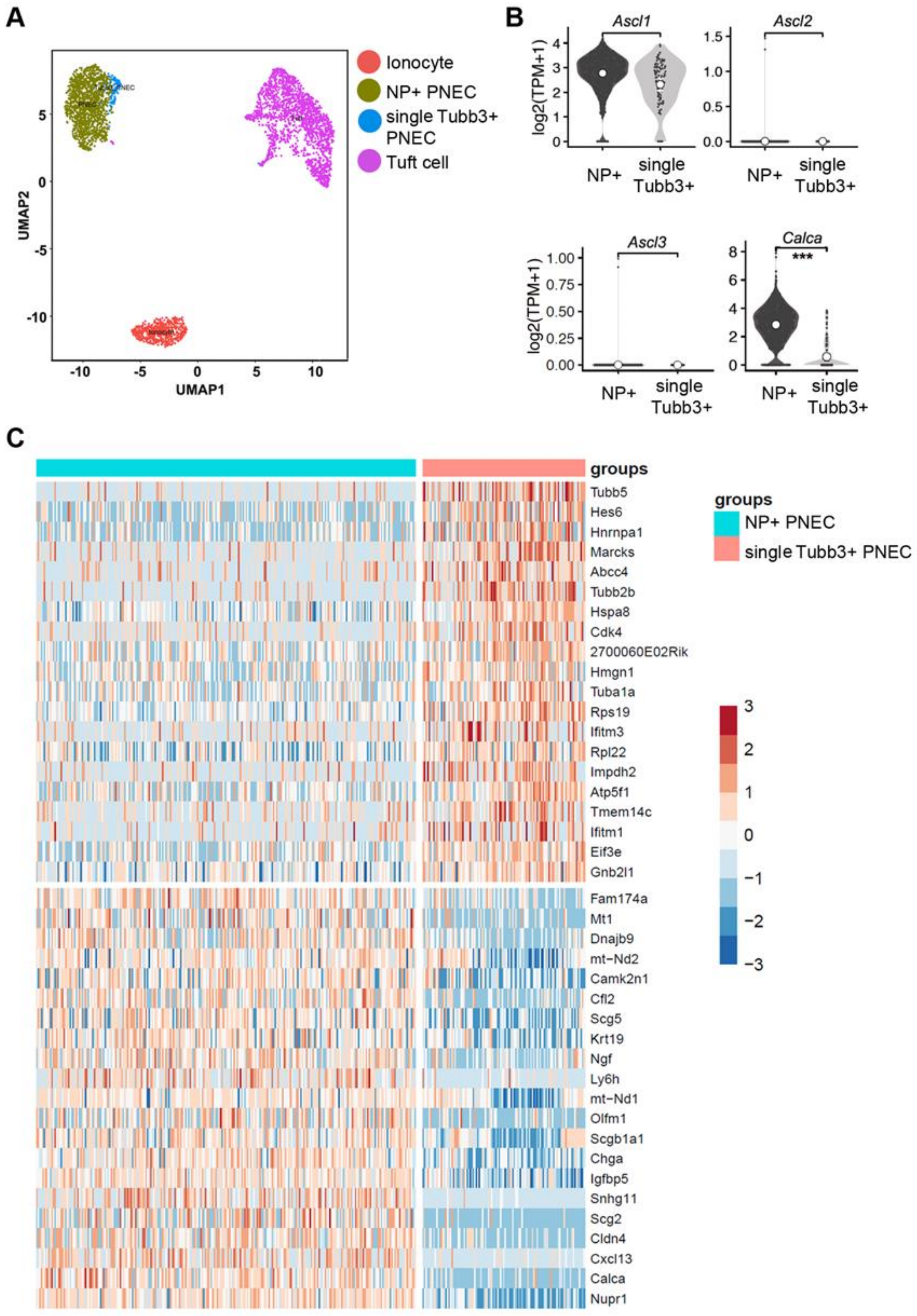

Figure S2. Heterogeneity of PNECs in the mouse trachea identified by single cell sequencing in a published study. Related to Figure 2. (**A**) Gene expression clusters by tSNE plot of the single cell

library from mouse trachea. The single cell RNA library was previously published (Montoro et al., 2018). For simplicity, gene clusters representing rare tracheal epithelial cell types including *Aslc1*$^+$ PNECs, *Ascl2*$^+$ tuft cells, and *Ascl3*$^+$ ionocytes were shown. PNECs that expressed neuropeptide genes were marked as NP$^+$ PNEC. PNECs that expressed only the *Tubb3* gene were marked as single Tubb3$^+$ PNEC. (**B**) Comparison of *Ascl1*, *Ascl2*, *Ascl3* and *CGRP* gene expression level between NP$^+$ PNECs and single Tubb3$^+$ PNECs. (**C**) Gene expression heatmap showing differentially expressed genes between NP$^+$ PNECs and single Tubb3$^+$ PNECs in the mouse trachea. ***p<0.001 by Student's t-test.

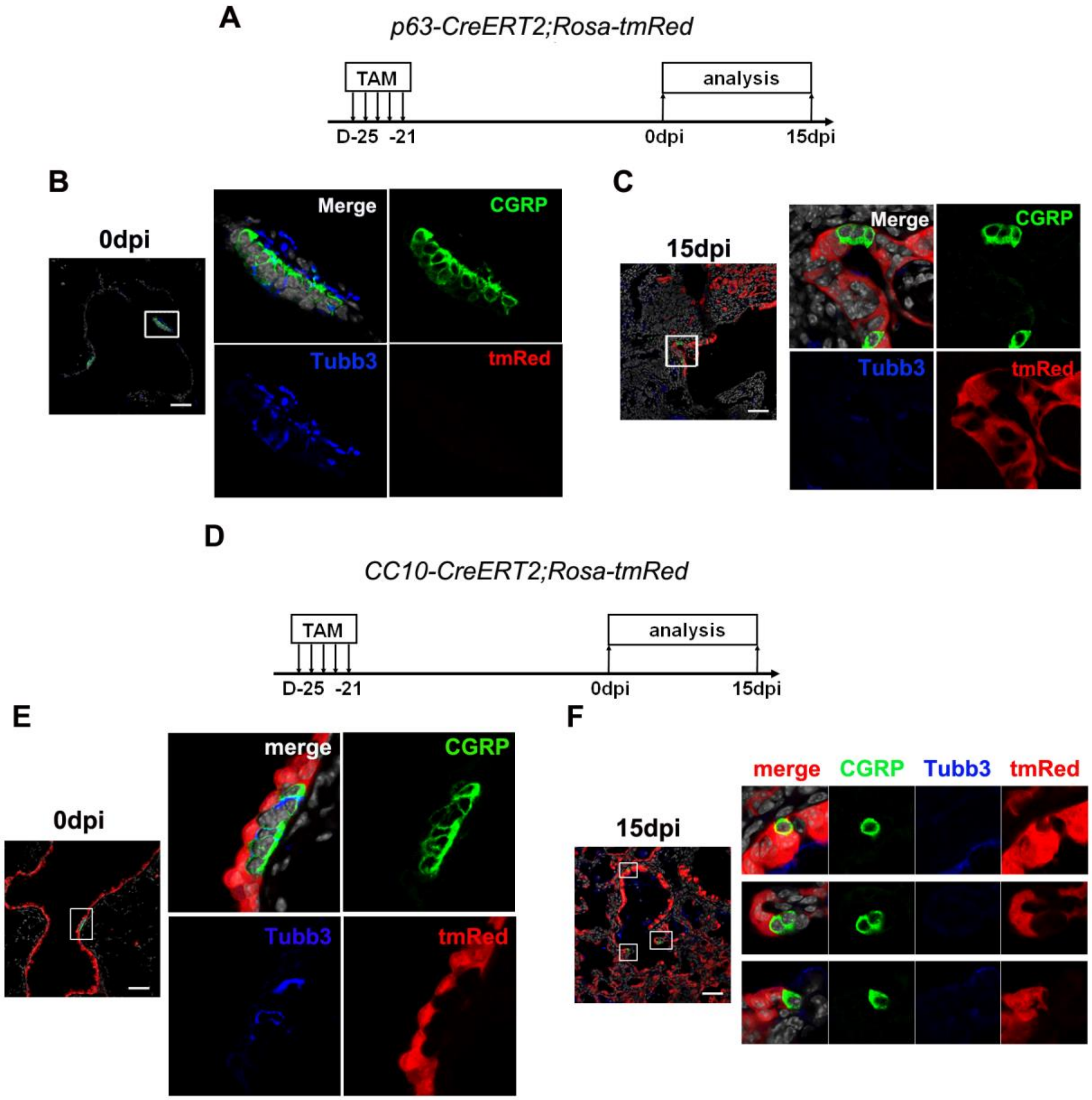

Figure S3. BSCs and club cells do not contribute to PNECs in the mouse intrapulmonary airway following H1N1 influenza viral infection. Related to Figure 3. (**A**) Scheme of BSC lineage tracing and viral infection in adult mice. The intrapulmonary airway was analyzed by double staining for CGRP and Tubb3 before infection (0 dpi) and 15 days after infection (15 dpi) (**B** and **C**). (**D**) Scheme of club cell lineage tracing in adult mice followed by influenza viral infection. The intrapulmonary airway was analyzed by double staining for CGRP and Tubb3 before and after infection (**E** and **F**). For all the panels of antibody staining, the outlined area is enlarged and shown by the color panels on the right. Nuclei were counterstained with DAPI. Data are representative results from 2 mice in each experimental group. Scale bars, 50 μm.

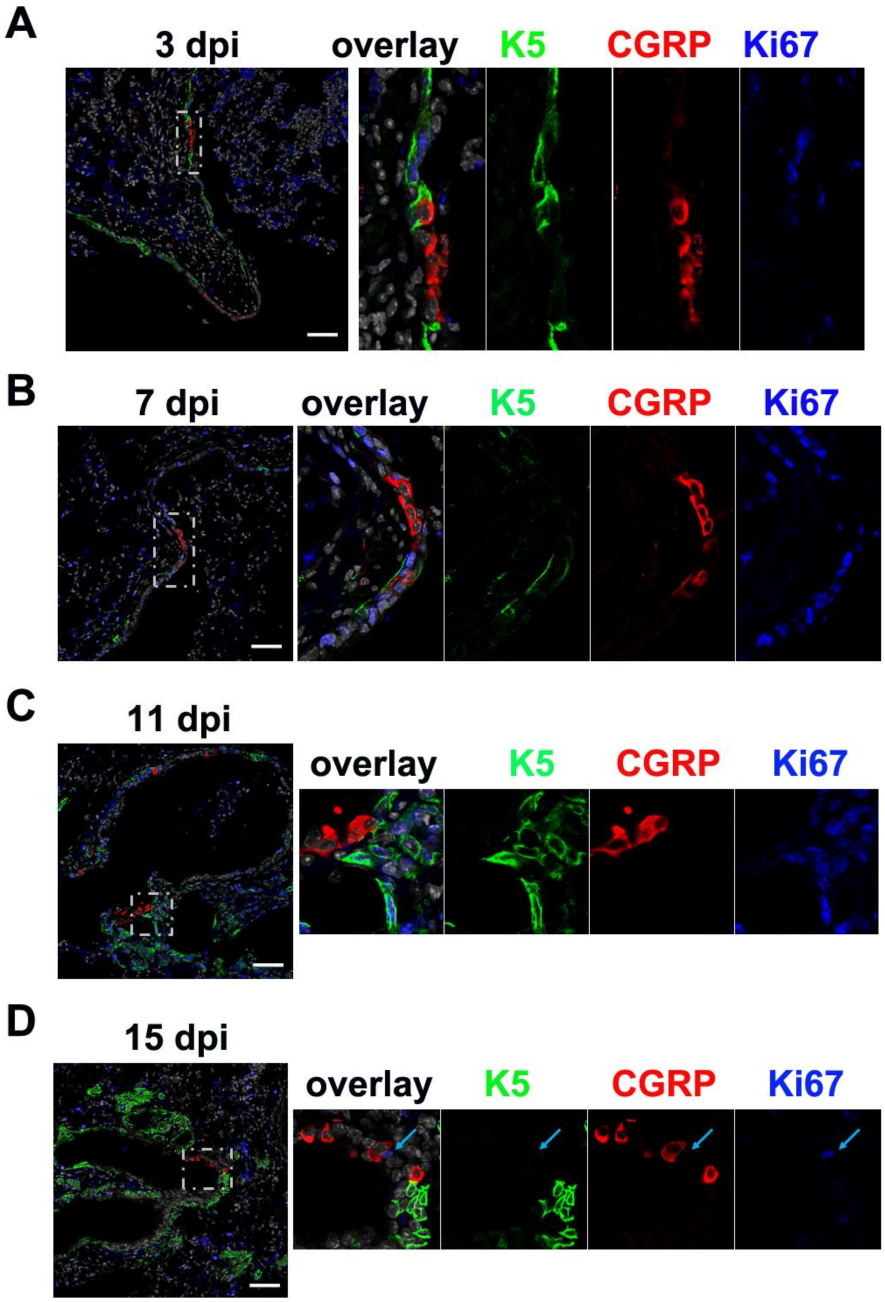

Figure S4. PNECs exhibit little proliferation following H1N1 influenza viral infection. Related to Figure 3. The mouse intrapulmonary airway was harvested at multiple time points following H1N1 infection. Tissue sections were stained for keratin 5 (K5), CGRP, and Ki67 to evaluate PNEC proliferation. The area outlined in the left panel was enlarged and shown by images on the right. Arrows in images at day 15 post infection point to one $Ki67^{+}$ cell that was $CGRP^{-}$. Data are representative results from 2 mice in each experimental group. Scale bars, 50 μm.

Supplementary Table 1: Related to Figure 1. Database of human tissues, cases, and controls.

**deceased donors of healthy lungs from IIAM**	**Age (years)**	**Medical Notes**
Donor #1	65	Male, died of stroke
Donor #2	53	Male, died of cardiovascular disease
Donor #3	43	Male, died of stroke
Donor #4	13	Female, died of head trauma
Donor #5	0	Female, asphyxiation
**Patient tissue (source)**	**Age (months)**	**Medical and Pathological Notes**
NEHI (BCH)	18 months	Chronic tachypnea and O_2 dependence since birth. Histologic sections show mild lymphocytic bronchitis/bronchiolitis and a probable increase in synaptophysin-positive neuroendocrine cells.
**Infant tissue (source)**	**Serum level of serotonin (ng/mL)**	**Notes**
Control #1 (SDME)	77.83	Premature birth (35 gestational weeks); postnatal respiratory distress; neonatal jaundice
Control #2 (SDME)	75.16	Aspiration pneumonia with chronic bronchiolitis
Control #3 (SDME)	23.37	Neonatal bradycardia and arrest with meconium aspiration
Control #4 (healthy donor #5, IIAM)	Not measured	Neonatal asphyxia
Control #5 (SDME)	78.20	**EXCLUDED** because the tissue section has no conducting airway.
Control #6 (SDME)	154.5	**EXCLUDED** because of the intermediate serum level of serotonin
SIDS, high serotonin #1, SDME)	414.0	Normal development
SIDS, high-serotonin #2 (SDME)	433.6	Premature birth (26 gestational weeks) with associated complications
SIDS, high serotonin #3 (SDME)	437.2	Normal development
SIDS, high serotonin #4 (SDME)	460.7	Normal development
SIDS, high serotonin #5 (SDME)	459.9	**EXCLUDED** because of postmortem autolysis
SIDS, normal serotonin #1 (SDME)	17.65	Premature birth (36 gestational weeks) with normal development
SIDS, normal serotonin #2 (SDME)	48.22	Normal development
SIDS, normal serotonin #3 (SDME)	41.38	Ureterocele and congenital hydronephrosis but with absence of infection or dehydration
SIDS, normal serotonin #4 (SDME)	39.85	Normal development
SIDS, normal serotonin #5 (SDME)	36.29	Normal development

Serum serotonin was determined via serotonin-specific ELISA and reported in Haynes et al 2017. IIAM, International Institute for the Advancement of Medicine. BCH, Boston Children's Hospital. SDME, San Diego Medical Examiner.
